# ALDsuite: Dense marker MALD using principal components of ancestral linkage disequilibrium

**DOI:** 10.1186/s12863-015-0179-y

**Published:** 2015-03-07

**Authors:** Randall C Johnson, George W Nelson, Jean-Francois Zagury, Cheryl A Winkler

**Affiliations:** BSP CCR Genetics Core, Leidos Biomedical Research, Inc, Frederick National Laboratory, Frederick, MD, 21702 USA; Chaire de Bioinformatique, Conservatiore National des Arts et Metieèrs, Paris, 75003 France; Basic Research Laboratory, Leidos Biomedical Research, Inc, Frederick National Laboratory, Frederick, MD, 21702 USA

**Keywords:** Admixture linkage disequilibrium, MALD, Admixture inference

## Abstract

**Background:**

Mapping by admixture linkage disequilibrium (MALD) is a whole genome gene mapping method that uses LD from extended blocks of ancestry inherited from parental populations among admixed individuals to map associations for diseases, that vary in prevalence among human populations. The extended LD queried for marker association with ancestry results in a greatly reduced number of comparisons compared to standard genome wide association studies. As ancestral population LD tends to confound the analysis of admixture LD, the earliest algorithms for MALD required marker sets sufficiently sparse to lack significant ancestral LD between markers. However current genotyping technologies routinely provide dense SNP data, which convey more information than sparse sets, if this information can be efficiently used. There are currently no software solutions that offer both local ancestry inference using dense marker data and disease association statistics.

**Results:**

We present here an R package, ALDsuite, which accounts for local LD using principal components of haplotypes from surrogate ancestral population data, and includes tools for quality control of data, MALD, downstream analysis of results and visualization graphics.

**Conclusions:**

ALDsuite offers a fast, accurate estimation of global and local ancestry and comes bundled with the tools needed for MALD, from data quality control through mapping of and visualization of disease genes.

## Background

It is well established that a subset of disease and trait phenotypes differ among human populations. Observed differences between ancestral groups can be attributed to two general causes: a difference in environmental exposures or factors or a difference in underlying genetic composition. Individuals with mixed ancestry provide an effective way to map phenotype/genotype associations to specific loci for diseases that show population-specific prevalence differences not fully explained by environmental factors [1,2]. When two populations combine to form a new admixed population, large chromosomal segments from each of the ancestral populations remain in circulation for many generations. The difference in allele and haplotype frequencies between the populations induces admixture linkage disequilibrium (ALD) that extends over much greater distances than the local LD inherited from ancestral populations. With each new generation chromosomes recombine and the extent of ALD becomes smaller, but with the sequencing of the human genome and the advances in genotyping technology of the last decade, the ancestral origin of chromosomal segments can be inferred with high accuracy for many generations post-admixture [3]. Admixture mapping using sparse SNP arrays have been used to identify the genetic bases for several traits and diseases, including renal disease, white blood count, and chronic obstructive pulmonary disease in African Americans [4-6].

The application of ALD information to gene mapping studies, also referred to as Mapping by Admixture Linkage Disequilibrium (MALD), is a statistically powerful method to identify genetic associations with disease in admixed populations when there is a difference in disease risk among ancestral groups not attributable to environmental factors [7]. The key advantage of this approach over the standard genome wide association study (GWAS) approach is that the effective number of statistical comparisons, for associations between markers and disease, is inversely related to the length of LD between markers and the causal disease locus. In African Americans, for example, ALD between loci as distant as 20 cM has been identified, while LD in non-admixed populations rarely extends longer than 0.1 cM [8,9]. This increases the power over classical GWAS by drawing focus to a specific region of interest with 200-500 fold fewer comparisons that must be corrected using multiple comparisons techniques [9].

As computational power has increased and the cost of genotyping and sequencing has decreased, MALD studies have become more common and successfully applied to identify a number of genetic variants associated with common diseases [4]. Several software packages, ADMIXMAP, ANCESTRYMAP and STRUCTURE, provided good estimates of global ancestry (i.e. the proportion of ancestors from each admixing population for an individual), as well as statistics for association between phenotype and local ancestry (i.e. the population each haplotype was inherited from at a particular locus) [10-12]. These early software packages were limited in their ability to analyze dense marker sets, due to their reliance on the lack of local LD among sampled markers. This reliance on sparse marker sets results from the additional complexity involved with the modeling of local LD. An attempt was made in one software package, SABER, to model 2-way LD of a marker with its immediate neighbors, but this was later shown to allow bias into the model from higher order local LD with more distantly linked markers [13,14]. The consequences of this bias include a tendency to overestimate the divergence of admixing populations and possible inference of significant admixture in unadmixed individuals [3].

Two recent software packages, HAPAA and HAPMIX, have modeled local LD in a Bayesian framework similar to that used for genotype imputation, with very good results [15,16]. These methods, however, can be computationally intensive, especially with increasingly dense marker sets [3]. Other recent algorithms, including LAMP-LD, MULTIMIX and RFMix, have mainly focused on local ancestry inference using disjoint haplotype blocks [17-19] (see Table 1 for a list of all currently available ancestry inference software). While this approach is much more computationally efficient and scales well with increasing marker density, many regions do not segregate well into haplotype blocks. Additionally, most of these methods bin markers in an arbitrary way, including a pre-determined number of markers in each bin along the chromosome. This can lead to vastly differing window sizes.

**Table 1:** **Currently available admixture inference software**

**Software**	**Dense**	**MALD**	**>2**	**Cited**	**References**
	**markers**		**pops**		
STRUCTURE			*✓*	12427	[12,20,21]
ADMIXMAP		*✓*		201	[22]
ANCESTRYMAP		*✓*		361	[11]
FRAPPE	*✓*		*✓*	255	[23]
SABER+	*✓*		*✓*	157	[13,24]
LAMP-LD	*✓*		*✓*	131	[17]
HAPAA	*✓*		*✓*	48	[15]
SWITCH-MHMM	*✓*		*✓*	35	[25]
WINPOP	*✓*		*✓*	54	[26]
HAPMIX	*✓*			210	[16]
ADMIXTURE	*✓*		*✓*	293	[27]
PCAdmix	*✓*		*✓*	14	[28]
MULTIMIX	*✓*		*✓*	9	[18]
SEQMIX	*✓*				[29]
ALDER	*✓*		*✓*	20	[30]
RFMix	*✓*		*✓*	7	[19]
ALLOY	*✓*		*✓*	1	[31]
EILA	*✓*		*✓*	2	[32]
DBM-Admix	*✓*		*✓*		[33]
MaCH-Admix	*✓*		*✓*	14	[34]
ELAI	*✓*		*✓*	2	[35]

Analysis of dense marker data, inclusion of disease association statistics, number of supported populations and number of citations listed on GoogleScholar as of August 14, 2014 are listed.

With the R package described here we provide local ancestry estimates using a hidden Markov model (HMM) algorithm similar to that used by existing software [10-12], with higher order local LD modeled indirectly using principal components of neighboring markers in groups designed to maintain consistent window size in cM. Additional features not provided in most admixture software packages include MALD association statistics, quality control measures and data formatting tools. Followup statistical and graphical analysis using the powerful tool set available in R is readily available.

## Implementation

### Principal component regression

**Figure 1:**
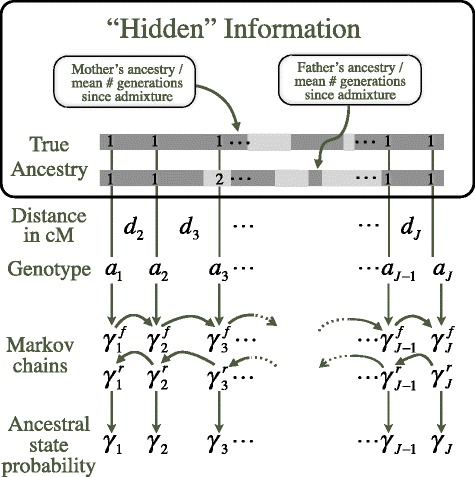
Hidden Markov Model for ancestry inference. Each individual’s local ancestral state probability, *γ*, is modeled as a function of preceding ancestral state probabilities in each Markov chain, genetic distance to neighboring markers, *d*, individual global ancestry parameters and observed haplotype or genotypes, *a*, in a region

We use a Hidden Markov Model (HMM) to model switches between ancestral states across each individual’s chromosomes (see Figure 1). The genome is split into equally sized windows (default is 0.1 cM), and an analysis of modern-day representatives of ancestral populations (e.g. West Africans and Europeans for evaluation of African American individuals), is used to obtain starting values for the HMM priors. This is done with a phased data set such as that provided by the International HapMap Project [36], which can be found in the accompanying companion package, ALDdata. These methods can be extended to unphased data, but phased data are currently required by ALDsuite.

Higher order ancestral LD information is approximated in this method using principal components (PCs) of the surrounding, linked markers, and a principal component analysis (PCA) is performed. Samples from modern-day surrogate populations chosen to represent ancestral, admixing populations are analyzed in the PCA, and PCs accounting for 80% of the observed variation are chosen to model the likelihood of each ancestral population within each window. The transformation of the genotype data using the PC loadings from the *l*^*th*^ surrogate ancestral population is illustrated in Equation 1 where the principal component matrix for a window is the matrix multiplication of the phased haplotype matrix, *A* (one row per chromosome, one column per marker in the haplotype) with the eigenvector matrix, *v*:

(1) $$ \text{PC}_{l}\left(A \right) = Av_{l}.  $$

A logistic Principal Component Regression (PCR) is then performed to infer the likelihood of each ancestral state within each window as a function of these PCs, and the regression coefficients are used as starting points for local ancestral state probability calculation in the HMM. In the case of two ancestral populations, this simplifies to a logistic regression (see Equation 2); a multinomial logistic regression is used to model admixture between more than two populations (see Appendix).

(2) $$ \begin{aligned} \log \left(\frac{\text{P}\left(g = 1\left| A \right. \right)}{\text{P}\left(g = 0\left| A \right. \right)} \right) &= \boldsymbol{\beta} \cdot \text{PC}_{1}\left(A \right) + \varepsilon,\\ \text{P}\left(g = 0\left| A \right. \right) &=\frac{1}{1+e^{\boldsymbol{\beta} \cdot \text{PC}_{1}\left(A \right)}}, \\ \text{P}\left(g = 1\left| A \right. \right) &=1 - \text{P}\left(g = 0\left| A \right. \right), \end{aligned}  $$

where *g* indicates the proposed ancestral population the haplotype originated from. In sparsely sampled regions, where only one marker was sampled within the bounds of the window, observed alleles are used in the model instead of PCR.

### HMM algorithm

The HMM is an iterative, two-step process: in the first step, ancestral state probabilities, *γ*, are calculated for each individual in the sample at each window, followed in the second step by an update of the parameters on which *γ* is conditioned (see Figure 1). A basic overview is given here; complete details are given in the Appendix section.

We calculate ancestral state probabilities using a forward-backward algorithm similar to other admixture HMMs [10-12], but using the PC loadings discussed above to account for local LD. The ancestral state probabilities in each Markov chain (i.e. one starting at each end of the chromosome, called the forward and reverse chains) consist of the ancestral state probabilities defined in Equation 2, conditioned on the ancestral state probability of the previous marker in the chain and the likelihood of recombination between the two:

(3) $$ \begin{aligned} \gamma_{1} &= \text{P}\left(g_{1}\left| A \right. \right) \\ \gamma_{j} &= \text{P}\left(g_{j}\left| A \right. \right)*\left[ \text{P}\left(r_{j} \right)G + \left(1 - \text{P}\left(r_{j} \right) \right)\gamma_{j - 1} \right], \end{aligned}  $$

where *γ*_*j*_ is a vector of ancestral state probabilities for the *j*^*th*^ window, P(*g*|*A*) is defined in Equation 2, *G* is the global ancestry or proportion of the genome inherited from each ancestral population, and P(*r*) is the probability of recombination between the midpoints of the current and previous windows. These probabilities are further dependent on the number of generations since admixture, and the genetic distance between window midpoints, *d*. The product of the forward and reverse Markov chains, *γ*_*f*_ and *γ*_*r*_, is normalized (so that they sum to one) to obtain the final ancestral state probabilities for each window, conditional on admixture linkage disequilibrium with nearby windows,

(4) $$ \gamma =\left\Vert {\gamma}^f\ast {\gamma}^r\right\Vert \kern1em . $$

The local ancestral state at each window is sampled using these ancestral state probabilities. Parameters informing the HMM, particularly those on which *γ* is conditioned (e.g. PCR coefficients in Equation 2, estimated global ancestry and estimated number of generations since admixture), are updated at the conclusion of each iteration, using the sampled ancestral states discussed in the preceding paragraphs (see Appendix section for more details).

ALDsuite retains computation efficiency as the number and density of markers increases by analyzing PCs of small chromosomal regions. Additional computational efficiency can be achieved in multicore environments with support for the parallelization of ALDsuite using a distributed MCMC approach in which a separate analysis, or chain, is run for each parallel process [37,38]. In order to avoid unnecessary duplication of effort during the burn-in phase, each chain reports back to the main process after each iteration, where a remote proposal of each parameter is calculated based on the average of all parallel chains. Each chain then updates its own local parameter space using a weighted sum of the local and remote proposals:

(5) $$ {\fontsize{8.5}{8}\begin{aligned} \frac{iter}{n\ burn }~*~local\ proposal~+~\left(1 - \frac{iter}{n\ burn} \right)~*~remote~proposal, \end{aligned}}  $$

where *iter* is the current iteration and *nburn* is the total number of burn-in iterations. This results in a quicker convergence to the equilibrium distribution while allowing each chain to start sampling at an independent state.

### Error checking

#### Marker checks

Several quality control checks can be performed on each marker using ALDsuite to identify potential genotyping errors, mapping errors, flipped markers and irregular variations in allele frequency:

1. Hardy-Weinberg Equilibrium is tested using the hwexact() function in the hwde package [39].
2. Markers with a missing data rate exceeding a user-defined threshold are screened (default threshold is 5%).
3. Allele frequencies from genotypic data coded as A/C/T/G are compared among populations to identify potential A-T/G-C flips that may have occurred in data originating from different sources. The default is to drop these markers from the analysis set.
4. Allele frequencies in the admixed population are compared with modern-day, ancestral surrogate population allele frequencies to identify potentially irregular loci.

#### Individual checks

ALDsuite also includes several quality control checks for individuals, to identify potentially bad samples which the user may wish to remove:

1. Individuals with a missing data rate exceeding a user-defined threshold are screened (default threshold is 5%).
2. When sex chromosome data are available, simple gender checks are performed and possible issues are flagged.
3. The sample is screened for potentially related individuals, and matches are flagged.

#### Parameter checks

The parameter state space can be saved at each iteration during the analysis for evaluation of convergence.

1. A function is provided to graphically display the desired parameters over the course of the burnin and follow-on phases of the analysis. Greater parameter variability can be expected during the burnin phase, and multiple MCMC chains can be compared to evaluate how variable parameters are across independent chains. Parameters who’s mean values change significantly during the follow-on phase indicate the need for a longer burnin phase.
2. To evaluate the representativeness of chosen modern-day surrogate samples, the value of *τ* should be checked (see Appendix section for more details). Higher values indicate a better fit; instances where *τ*<50−100 either indicate poorly chosen modern-day surrogates or the presence of allele flips. In the analysis of African American data, using the YRI and CEU HapMap data as modern-day surrogate samples, we have observed *τ*∈(200−1000), depending on the density of the marker set.

### Statistical association

Local and global ancestry estimates across the genome are reported for each individual. With this information the user can use one of several statistical association techniques for mapping disease genes and/or fine mapping of disease-associated loci. When mapping disease genes by ALD, an association with local ancestry at a locus is the primary association being tested. The case-only regression model, defined in Equation 6, compares the difference between local ancestry and global ancestry. Other data (e.g. case-control) can be similarly modeled as defined in Equation 7. In both of these models, regions with statistically significant regression coefficients for local ancestry are inferred to harbor disease modifying genes.

(6) $$ \text{global ancestry} \sim \beta_{0} + \beta_{1}(\text{local ancestry}) + \beta_{2}(\text{covariates})   $$

(7) $$ \begin{aligned}  link(Y) & \sim \beta_{0} + \beta_{1}(\text{local ancestry}) + \beta_{2}(\text{global ancestry}) \\ & \quad +\beta_{3}(\text{covariates}) \end{aligned}  $$

When a disease locus is identified, a fine mapping analysis is needed to identify specific variants most strongly associated with the disease outcome. In a fine mapping analysis both ancestral and genotype data are included in the model (see Equation 8), and an association between genotype and disease is the primary association being tested.

(8) $$ \begin{aligned} link(Y) & \sim \beta_{0} + \beta_{1}(\text{genotypes}) + \beta_{2}(\text{local ancestry}) \\ & \quad + \beta_{3}(\text{global ancestry}) + \beta_{4}(\text{covariates}) \end{aligned}  $$

These generalized linear models are very flexible, allowing for multiple types of disease phenotypes (e.g. continuous, dichotomous, time-to-event) and any covariates deemed appropriate by the investigator. Wrapper functions for these models along with support for parallel computation is included in ALDsuite.

### Simulations and power

#### Control populations

Chromosomes with known ancestry at each marker were simulated in a two step process: 1) recombination points were assigned to each chromosome based on the number of generations since admixture; 2) chromosomal segments were randomly selected from the YRI and CEU HapMap samples to fill in each chromosomal region, with the probablility of sampling a given HapMap chromosome conditional upon the assigned global ancestry for the simulted chromosome. In this way, admixed chromosomes were simulated with appropriate admixture linkage patterns across the chromosome without regard to how windows are chosen.

Random recombination rates, conditional upon the number of generations since admixture, and global ancestral proportions, *G*, were sampled, and 400 chromosomes were simulated. Values for the number of generations since admixture were Gamma distributed with a mean of 6 and standard deviation of 2, and values for *G* were Beta distributed with a mean of 0.82 and standard deviation of 0.1. These parameters were chosen to simulate a typical African American sample. The CEU and YRI populations were also used as modern-day representative populations, but with the initial PCR estimates randomly modified to simulate imperfect surrogates. This was done by adding a normal random value to each of the regression estimates, the variance of which was scaled by each estimate’s standard error.

A sample of 100 individuals from each simulation above was analyzed using ALDsuite, MULTIMIX and PCAdmix [18,28], and the proportion of correct and incorrect inferences are reported.

### Empirical data

The ASW population from the International HapMap Project, a sample of African Americans from the Southwest USA, were analyzed using YRI and CEU populations as surrogate ancestral populations. These populations were analyzed using ALDsuite as well as MULTIMIX and PCAdmix [18,28], and a representative sample of the results on chromosome 20 are shown.

### Additional tools

Several tools are included in the R package, additional to the local ancestry inference and disease association statistics described above. These include input and output data formatting aids, quality control and analysis of the data, and useful data sets. Formatting functions are available for generating prior parameter estimates for different populations using HapMap populations contained in the ALDdata package, and calculation of genetic distance in humans is performed using one of several maps, including the International HapMap Project and those generated by Matise et al. [36,40,41]. Error checking functions for quality control measures discussed in the Error Checking section are included as well as some basic graphics. Additional downstream statistical analysis and custom generation of graphics using the diverse and powerful toolset provided by R is also directly available [42].

## Results and discussion

While sparse marker panels are more cost effective and have proven powerful in the detection several important disease risk genes, dense data provide more accurate ancestry inference and a finer resolution of recombination points [13]. One strategy that has been used is to follow up a MALD study with fine typing around an associated locus [43]. With ALDsuite both sparse and dense marker data are analyzed in combination, resulting in better global ancestry estimates, while being able to infer local ancestry on a much finer scale in areas of particular interest. This program should increase the utility of dense marker datasets available from many large cohort studies that include African Americans.

**Figure 2:**
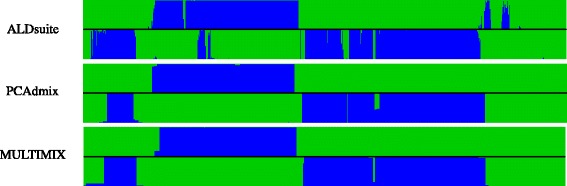
Representative chromosomes from one individual in the ASW population. Local ancestry inference along chromosome 20 is shown for ALDsuite (top), PCAdmix (middle) and MULTIMIX (bottom). A stacked bar plot indicating the inferred probability of African ancestry (represented by green bars) and European ancestry (represented by blue bars) is given for each phased haplotype. The width of each bar is proportional to the window size (in cM) covered by the markers used to infer ancestry

ALDsuite provides accurate inference of local ancestry, while indirectly modeling local, higher order LD remaining from ancestral populations. The analysis of our simulation resulted in 96.3% accuracy of local ancestry inference, compared to the 98.1% accuracy of PCAdmix and the 98.7% accuracy of MULTIMIX, which is on par with other leading analysis software [16,32]. Comparison of chromosomes from an analysis of the ASW population using ALDsuite, MULTIMIX and PCAdmix also shows a good degree of concordance between the methods used (see Figure 2).

One striking difference between the results shown in Figure 2 are the differing window sizes. The binning of markers in MULTIMIX and PCAdmix is done by arbitrarily grouping a fixed number of markers into each bin. In more densely sampled areas, such as those closer to the center of the chromosome, the window sizes are quite small, while other less densely sampled areas have much larger window sizes. The region at the beginning of the chromosomes in Figure 2, for example, cover as much as 4 cM. Binning of markers in ALDsuite is done by genetic distance, rather than the number of markers, creating a more constant window size across the genome. In more densely sampled regions, this helps maintain better computational properties, since fewer windows can be used to cover the same region, while in sparsely sampled regions a more precise estimate of the boundaries of ancestral haplotypes can be obtained.

Another key feature of ALDsuite that all other dense-marker admixture software lacks is direct access to statistical methods needed to map disease phenotypes. Not only does ALDsuite provide utilities directly supporting admixture mapping and fine mapping studies (see Implementation section), but many other proposed methods can be easily implemented in R, using the output provided by ALDsuite [44-46]. Also, of eleven MALD studies published in 2013 and early 2014, six used sparse marker panels for disease gene mapping, at least two of which explicitly thinned their dense marker data to accommodate the software used [47,48]. An additional 15 GWAS studies we identified from 2013 used various software listed in Table 1 to control for population substructure resulting from admixture, mostly using dense marker strategies (citations not listed here). This trend highlights the need for a dense marker software package that, like most sparse marker software, includes disease association statistics for MALD.

## Conclusion

Admixture inference software can be categorized using a few different metrics including the number of admixing populations it can simultaneously infer, the way it models local LD when analyzing dense marker data, the number of admixing populations it will simultaneously infer and support of disease gene mapping (see Table 1). There are currently no software solutions which both offer analysis of dense marker data from more than two admixing populations and disease association statistics, requiring the use of several software programs, often with very different input and output data formats. ALDsuite offers a fast, accurate estimation of global and local ancestry with the tools needed from data quality control through mapping of disease genes, along with the rich statistical and graphical utilities provided with R.

## Availability and requirements

**Project name:** ALDsuite **Project homepage:**https://github.com/johnsonra/ALDsuite**Operating systems(s):** Windows, Mac, Linux **Programming language:** R and C **Other Requirements:** R, version 3.0 or greater with the parallel, mvtnorm and hwde packages installed. The gdata and ncdf R packages are also recommended. **License:** GPL

## Appendix

## ALDsuite: Dense marker MALD using principal components of ancestral linkage disequilibrium

Computational details for the algorithm used to sample the joint distribution of the HMM for inferring local ancestry. Throughout, parameters are indexed by *i* (individual), *j* (marker), *c* (chromosome) and *k* (ancestral population).

## Initialization of the parameter space

Distances, *d*, are calculated as the number of centimorgans to the previous marker, with each chromosome starting with a missing value.

The modern allele frequencies on chromosome segments originating from ancestral populations, *Ω*, parameterize the prior distribution of ancestral allele frequencies, *P*. Eigen vectors for groups of markers used in modeling of ancestral LD within each ancestral population are either given by the user or estimated from HapMap data by the software. Prior estimates of logistic regression coefficients, *H*, and their associated variance-covariance matrices, *Σ*, for inference of modern allele frequencies as a function of nearby, linked markers are also either provided by the user or estimated from HapMap data. All associated markers within a user definable window (default is 2 cM) are chosen to model ancestral LD, and the number of principal components, *m*-1, accounting for 80% of the genetic variation in each subset are chosen to be included in the model, making a total of *m* coefficients, including the intercept.

Initial values for ancestry, *A*, are obtained using a quick frequentist algorithm, and global ancestry estimates for each parent are initially equal.

Initial values for average number of generations since admixture, *λ*, and effective population size of each prior population, *τ*, can also be specified by the user. When unspecified, default values tuned to the analysis of African Americans are used.

## MCMC Algorithm

### Step 1. Sample Ancestral States

Ancestral state probabilities are calculated using a forward-backward algorithm similar to that used by admixture software for sparse marker sets [10-12]. The main differences in our algorithm being that ancestral LD is indirectly modeled, allowing analysis of dense marker sets, and we estimate marginal ancestral state probabilities for each inherited chromosome, requiring the genotype data to be phased prior to analysis. These differences motivate the majority of differences between our package and other admixture software. In the forward portion of the algorithm, ancestral state probabilities, *γ*, are calculated at each locus, dependent on the genotype at each locus (probability that the *i*th individuals *j*th locus of chromosome *c* originated from the *k*th ancestral population).

(A1) $$ \gamma = \left\{ \begin{array}{ll} \frac{\text{P}\left(a = x | g = k \right) \text{P}\left(g = k \right)}{\text{P}\left(a = x \right)} &, a\ \text{known} \\ \text{P}\left(g = k \right) &, a\ \text{unknown} \end{array} \right.   $$

Before we treat the probabilities in Equation A1, we note that the probability of an observed recombination event, *r*_*ijc*_, over a distance of *d*_*j*_ cM is a function of the number of generations since admixture, *λ*_*ic*_:

(A2) $$\begin{array}{@{}rcl@{}} \text{P}\left(r | \lambda = 1 \right) & = & \frac{1 - e^{-2d/100}}{2},  \end{array} $$

(A3) $$\begin{array}{@{}rcl@{}} \text{P}\left(r \right) & = & 1 - \left(\frac{1 + e^{-2d/100}}{2} \right)^{\lambda}  \end{array} $$

and the probability of any crossovers happening in one haplotype since admixture over a window of size *w* cM follows a Poisson distribution:

(A4) $$ \text{P}\left(X > 0 | w \right) = 1 - e^{-\lambda w / 100}.  $$

The probability of an individuals genotype at a locus, *a*, conditional on the ancestral state, *g*, is a function of the allele frequencies in each population and the principal components of nearby, linked markers, spanning a region of *w* cM.

(A5) $$\begin{array}{@{}rcl@{}} \text{P}\left(a = x | a_{\bullet}, g = k \right) & = & \left\{ \begin{array}{ll} 1 - f_{j}\left(a_{\bullet}, k, w \right) &, x = 0 \\ f_{j}\left(a_{\bullet}, k, w \right) &, x = 1 \end{array} \right.  \end{array} $$

(A6) $$\begin{array}{@{}rcl@{}} f_{j}\left(a_{\bullet}, k, d \right) & = & p*\text{P}\left(X > 0 | w \right) + \\ & & \text{logit}^{-1}\left(\beta_{0} + \beta_{1}\text{PC}_{1}\left(a_{\bullet} \right) + \cdots \right)\\&& \left(1 - \text{P}\left(X > 0 | w \right)\right),  \end{array} $$

where the probability of one or more crossovers in the haplotype block of *w* cM, which informs the principal components regression, is defined in Equation A3, and *p*_*jk*_ is the allele frequency in chromosomes with *k* ancestry. We highlight the dependence of Equation A5 on the probability of observing crossovers within the window supporting the principal components regression. If there is a crossover, the resulting haplotype is no longer representative of the ancestral population, and we rely upon the allele frequency instead.

The probabilities of each ancestral state are further dependent on the ancestral probabilities at the previous locus, *γ*_*i*(*j*−1)*K*_, the distance, *d*_*j*_, between these loci (missing if it is the first locus on a chromosome), the individuals recombination rates, *λ*_*ic*_, and the individuals global ancestry, *A*_*ick*_ (the distance between loci is in cM).

Now we treat the probability of the ancestral state, *k*, of a locus, dependent on the ancestral state at the previous locus in the Markov chain, *k*^∗^:

(A7) $$ \text{P}\left(g = k \right) = A*\text{P}\left(r \right) + \gamma_{j-1} * \left(1 - \text{P}\left(r \right)\right).   $$

For the first locus on each chromosome, the only prior information available is the global ancestry of the parents. We essentially treat this scenario as if there were a known recombination event, i.e. P(*r*_1_)=1.

This also applies to the marginal probability of the observed genotype, *a*, which depends Equation A4 and Equation A7:

(A8) $$ \text{P}\left(a = x \right) = \sum_{k} \text{P}\left(a = x | g = k \right)\text{P}\left(g = k \right).   $$

The reverse chain is nearly identical, starting from the opposite end of each chromosome and working back. The final probabilities at each locus are obtained by multiplying the forward and reverse chains and normalizing,

(A9) $$ \gamma = \left\lVert \gamma^{f} * \gamma^{r} \right\rVert,   $$

and a sample, *G*, of *γ* is taken for use in Step 2:

(A10) $$ G \sim \text{Multinomial}\left(\gamma \right).   $$

### Step 2: Parameter Updates

#### Updates of A and A ^*X*^, global ancestry

The prior of *A* is Dirichlet distributed and parameterized by *ω*. The posterior is Dirichlet distributed, parameterized by the sum of *ω* and *γ*, for all autosomal markers.

(A11) $$\begin{array}{@{}rcl@{}} {}A & \sim & \text{Dirichlet}\left(\omega_{1}, \ldots, \omega_{K} \right)  \end{array} $$

(A12) $$\begin{array}{@{}rcl@{}} {}\dot{A} & \sim & \text{Dirichlet}\left(\omega_{1} + \sum_{jc}\gamma_{1}, \ldots, \omega_{K} + \sum_{jc}\gamma_{K} \right)  \end{array} $$

We accept the sampled values for each Metropolis-Hastings sample, $\dot {A}$, with probability

(A13) $$ \text{min}\left(1, \frac{\sideset{}{_{k}}\prod \dot{A}^{\omega-1}}{\sideset{}{_{k}}\prod A^{\omega-1}} \right).   $$

Patterson et. al. [11] have noted that sex chromosome ancestry is highly correlated with autosomal chromosome ancestry. Sex chromosome ancestry proportions are parameterized the same way here, by a scalar value, *omega*_*X*_, conditional on *A*. The posterior is Dirichlet distributed, parameterized by the product of *A* and *ω*^*X*^ and the sum of *γ* over the X chromosome.

(A14) $$\begin{array}{@{}rcl@{}} {} A^{X} & \sim & \text{Dirichlet}\left(\omega^{X} A \right)  \end{array} $$

(A15) $$\begin{array}{@{}rcl@{}} {}\dot{A}^{X} & \sim & \text{Dirichlet}\left(\omega^{X} A_{1} + \sum_{jc}\gamma_{1}, \ldots, \omega^{X} A_{K} + \sum_{jc}\gamma_{K} \right)  \end{array} $$

We accept the sampled values for each Metropolis-Hastings sample, $\dot {A}^{X}$, with probability

(A16) $$ \text{min}\left(1, \frac{\sideset{}{_{k}}\prod \left(\dot{A}^{X} \right)^{\omega^{X} A_{k} - 1}}{\sideset{}{_{k}}\prod \left(A^{X} \right)^{\omega^{X} A_{k} - 1}} \right).   $$

#### Update of *λ*, mean number of generations since admixture

The prior of *γ* is Gamma distributed, parameterized by a shape parameter, *α*_1_ and a rate parameter, *α*_2_.

(A17) $$ \lambda \sim \text{Gamma}\left(\alpha_{1}, \alpha_{2} \right)   $$

The posterior is Gamma distributed:

(A18) $$ \dot{\lambda} \sim \text{Gamma}\left(\alpha_{1} + \#\ crossovers, \alpha_{2} + \sum_{j} d \right).   $$

As noted in Equation A3, the number of crossovers is Poisson distributed. To sample the number of crossovers in each individual, conditional on there being at least 1 crossover, we generate a random uniform number for each locus, *q*_*j*_, such that

(A19) $$ q_{j} \in \left(\text{P}\left(x = 0; \lambda_{ic}, d_{j} \right), 1 \right)   $$

and the number of corresponding crossovers for each locus, *n*_*xj*_, such that

(A20) $$ \text{P}\left(x = nx_{j} - 1; \lambda_{ic}, d_{j} \right) < q_{j} \leq \text{P}\left(x = nx_{j}; \lambda_{ic},d_{j} \right).   $$

We then calculate the probability of 0 crossovers given *G*, *px*_*j*0_, at each locus,

(A21) $$\begin{array}{@{}rcl@{}} px_{j0} & = & \text{P}\left(x = 0 \mid G_{ic}; \lambda_{ic}, d_{j} \right)  \\ & = & 1 - \text{P}\left(x > 0 \mid G_{ic}; \lambda_{ic}, d_{j} \right)  \end{array} $$

where

(A22) $${} {\small{\begin{aligned} \text{P}\left(x > 0 \mid G_{ic}; \lambda_{ic}, d_{j} \right) = \left\{\!\! \begin{array}{cl} 1 &, g_{ijc} \neq g_{i(j - 1)c} \\ \frac{A_{icg}\left(1 - e^{-\lambda_{ic}d_{j}} \right)}{e^{-\lambda_{ic}d_{j}} + A_{icg}\left(1 - e^{-\lambda_{ic}d_{j}} \right)} &, g_{ijc} = g_{i(j-1)c} \end{array} \right.\!. \end{aligned}}}  $$

We keep the number of crossovers we sampled, *nx*_*j*_, at that locus with probability 1−*px*_*j*0_. The sum of these sampled crossovers, we can sample the updated value, $\dot {\lambda }$, which we keep with probability

(A23) $$ \text{min}\left(1, \frac{\dot{\lambda}^{\alpha_{1} - 1} e^{-\alpha_{2} \dot{\lambda}}}{\lambda^{\alpha_{1} - 1} e^{-\alpha_{2} \lambda}} \right).   $$

#### Updates of *p* and *β*, parameterizing allele frequencies for each population

The prior allele frequency of *p* is Beta distributed, parameterized by the product of *τ* and *P*. The posterior is Beta distributed, parameterized by sum of the product of *τ* with *P* and the number of reference/variant alleles sampled in Step 2.

(A24) $$\begin{array}{@{}rcl@{}} p & \sim \text{Beta} & \left(\tau P, \tau (1 - P) \right)  \end{array} $$

(A25) $$\begin{array}{@{}rcl@{}} \dot{p}_{jk} & \sim \text{Beta} & \left(\tau_{k} P_{jk} + \#\ reference\ alleles, \right. \\ & & \left. \tau_{k} \left(1 - P_{jk} \right) + \#\ variant\ alleles \right)  \end{array} $$

Each proportion is individually updated and is kept with probability

(A26) $$ \text{min}\left(1, \frac{\sideset{}{_{ic}}\prod \dot{p}^{\tau P-1} (1 - \dot{p})^{\tau(1 - P)-1}} {\sideset{}{_{ic}}\prod p^{\tau P-1} (1 - p)^{\tau(1 - P)-1}} \right).   $$

For principal component regression modeling of the allele probabilities, conditional on local ancestry, *β* is multivariate normally distributed, parameterized by the prior *B* and the diagonal of *Σ*. The posterior is additionally parameterized by *τ* and the logistic regression coefficients, $\hat {\beta }$, of the principal component regression model of the haplotypes sampled at the end of Step 1.

(A27) $$\begin{array}{@{}rcl@{}} \beta & \sim & \text{N}\left(B, \frac{1}{\tau^{2}} \text{diag}\left(\Sigma \right) \text{I} \right)  \end{array} $$

(A28) $$\begin{array}{@{}rcl@{}} \dot{\beta} & \sim & \text{N}\left(\frac{n\hat{\beta} + \tau B}{n + \tau}, \frac{1}{\left(n + \tau \right)^{2}} \text{diag}\left(\Sigma \right) \text{I} \right)  \end{array} $$

The sampled value, $\dot {\beta }$, is kept with probability

(A29) $${} {\small{\begin{aligned} \text{min}\left(1, e^{\frac{-\tau^{2}}{2}\left[ \left(\dot{\beta} - B \right)^{T} \left(\text{diag} \left(\Sigma \right) \text{I} \right)^{-1} \left(\dot{\beta} - B \right) - \left(\beta - B \right)^{T} \left(\text{diag} \left(\Sigma \right) \text{I} \right)^{-1} \left(\beta - B \right) \right]} \right)\!. \end{aligned}}}  $$

#### Update of *P*, *B* and *τ*, hyper parameters for *p* and *β*

The prior of *P* is Beta distributed, parameterized by the number of observed alleles in the modern day equivalent to the founder populations (e.g. Africans and Europeans for African Americans).

(A30) $$ P \sim \text{Beta}\left(\Omega \right)   $$

*Ω* is a vector of the number of variant alleles and the number of reference alleles in the modern-day ancestral surrogate population sample. After each update of *P*, $\dot {P}_{\textit {jk}}$, the change is kept with probability

(A31) $${} {\small{\begin{aligned} \text{min}\left(1, \frac{\sideset{}{_{k}}\prod \Gamma(\tau P) \Gamma(\tau (1 - P)) p^{\tau \dot{P} - 1}(1 - p)^{\tau (1 - \dot{P}) - 1}} {\sideset{}{_{k}}\prod \Gamma(\tau P) \Gamma(\tau (1 - P)) p^{\tau P - 1}(1 - p)^{\tau (1 - P) - 1}} \right). \end{aligned}}}  $$

The prior of *B* is multivariate normally distributed as a function of *H* and *Σ*, as estimated from the modern-day surrogate ancestral population.

(A32) $$ B \sim \text{N}\left(H, \text{diag}\left(\Sigma \right) \text{I} \right)   $$

Sampled updates, $\dot {B}$, are kept with probability

(A33) $${} {\small{\begin{aligned} \text{min}\left(1, e^{\frac{-\tau^{2}}{2}\left[ \left(\beta - \dot{B} \right)^{T} \left(\text{diag} \left(\Sigma \right) \text{I} \right)^{-1} \left(\beta - \dot{B} \right) - \left(\beta - B \right)^{T} \left(\text{diag} \left(\Sigma \right) \text{I} \right)^{-1} \left(\beta - B \right) \right]} \right)\!. \end{aligned}}}  $$

The prior of *τ* is log normally distributed such that log _10_(*τ*) has a mean of 2 and standard deviation of 0.5,

(A34) $$ \text{log}_{10}(\tau) \sim \text{N}(2, 0.5).   $$

Samples values, $\dot {\tau }$, are kept with respective probabilities,

(A35) $$ \text{min}\left(1, LR\left(\dot{\tau}, \tau \mid p, P \right) * LR\left(\dot{\tau}, \tau \mid \beta, B \right)\right)   $$

where, given the length of *β*=*l*,

(A36) $${} {\small{\begin{aligned} LR\left(\dot{\tau}, \tau \mid p, P \right) = \frac{\sideset{}{_{k}}\prod \Gamma(\tau P) \Gamma(\tau (1 - P)) p^{\tau \dot{P} - 1}(1 - p)^{\tau (1 - \dot{P}) - 1}} {\sideset{}{_{k}}\prod \Gamma(\tau P) \Gamma(\tau (1 - P)) p^{\tau P - 1}(1 - p)^{\tau (1 - P) - 1}} \end{aligned}}}  $$

(A37) $${} {\small{\begin{aligned} LR\left(\dot{\tau}, \tau \mid \beta, B \right) & = & \prod_{jk} \left(\frac{\dot{\tau}}{\tau} \right)^{-l} e^{\frac{\tau^{2} - \dot{\tau}^{2}}{2} \left[ (\beta - B)^{T} \left(\text{diag}(\Sigma) \text{I} \right)^{-1} (\beta - B) \right]}. \end{aligned}}}  $$

#### Update of *ω* and *ω*^*X*^, hyper parameters for *A* and *A*^*X*^

The prior of *ω* and *ω*^*X*^ are log normally distributed, such that log _10_(*ω*) has mean 1 and standard deviation 0.5.

(A38) $$\begin{array}{@{}rcl@{}} \text{log}_{10}(\omega) & \sim & \text{N}(1, 0.5)  \end{array} $$

(A39) $$\begin{array}{@{}rcl@{}} \text{log}_{10}(\omega^X) & \sim & \text{N}(1, 0.5)  \end{array} $$

Updated values for *ω* and *ω*^*X*^, $\dot {\omega }$ and $\dot {\omega }^{X}$, are kept with probability

(A40) $$\begin{array}{@{}rcl@{}} {}\text{min}\left(1, \prod_{ic} \frac{\Gamma\left(\sideset{}{_{k}}\sum \dot{\omega} \right) \sideset{}{_{k}}\prod \Gamma(\omega) A^{\dot{\omega} - 1}} {\Gamma\left(\sideset{}{_{k}}\sum \omega \right) \sideset{}{_{k}}\prod \Gamma(\dot{\omega}) A^{ \omega - 1}} \right),  \end{array} $$

(A41) $$\begin{array}{@{}rcl@{}} {}\text{min}\left(1, \prod_{ic} \frac{\Gamma\left(\sideset{}{_{k}}\sum A\dot{\omega}^{X} \right) \sideset{}{_{k}}\prod \Gamma(A \omega^X) \left(A^{X} \right)^{A\dot{\omega}^{X} - 1}} {\Gamma\left(\sideset{}{_{k}}\sum A \omega ^{X} \right) \sideset{}{_{k}}\prod \Gamma(A\dot{\omega}^X) \left(A^{X} \right)^{A \omega ^{X} - 1}} \right).  \end{array} $$

#### Update of *α*, hyper parameters for *λ*

Similar to other admixture software, updates of are a function of the mean of the Gamma distribution, *α*_1_/*α*_2_=*m*, and the variance of the Gamma distribution, $\alpha _1/\alpha _{2}^{2} = v$. Each is log normally distributed, such that log _10_(*m*) and log _10_(*v*) each have mean 1 and standard deviation 0.5.

(A42) $$\begin{array}{@{}rcl@{}} \text{log}_{10}(m) & \sim & \text{N}(1, 0.5)  \end{array} $$

(A43) $$\begin{array}{@{}rcl@{}} \text{log}_{10}(v) & \sim & \text{N}(1, 0.5)  \end{array} $$

Values for *m* and *v* are updated independently, parameterized by $\dot {\alpha }$, and are kept with probability

(A44) $$ \text{min}\left(1, \frac{\sideset{}{_{ic}}\prod \Gamma(\alpha_1) \dot{\alpha}_{2}^{\dot{\alpha}_{1}} \lambda^{\dot{\alpha}_{1} - 1} e^{-\dot{\alpha}_{2}\lambda}} {\sideset{}{_{ic}}\prod \Gamma(\dot{\alpha}_1) \alpha_{2}^{\alpha_{1}} \lambda^{\alpha_{1} - 1} e^{-\alpha_{2}\lambda}} \right).  $$
